# The Impact of COVID-19 Vaccination in Changing the Adherence to Preventive Measures: Evidence from Italy

**DOI:** 10.3390/vaccines10050777

**Published:** 2022-05-13

**Authors:** Francesco Corea, Lucio Folcarelli, Annalisa Napoli, Grazia Miraglia del Giudice, Italo Francesco Angelillo

**Affiliations:** 1 Department of Experimental Medicine, University of Campania “Luigi Vanvitelli”, Via Luciano Armanni 5, 80138 Naples, Italy; francesco.corea@studenti.unicampania.it (F.C.); lucio.folcarelli@studenti.unicampania.it (L.F.); annalisa.napoli@studenti.unicampania.it (A.N.); grazia.miragliadelgiudice@studenti.unicampania.it (G.M.d.G.); 2 Department of Public Health and Laboratory Services, Teaching Hospital of the University of Campania “Luigi Vanvitelli”, Via Luciano Armanni 5, 80138 Naples, Italy

**Keywords:** COVID-19, vaccination, preventive measures, behaviors, attitude

## Abstract

The objectives of the survey were to explore any changes in the adherence to the three main COVID-19 preventive measures (social distancing, washing hands, wearing face-masks) among 795 individuals who received the COVID-19 vaccine booster dose in Italy and to identify the predictors associated. The concern of contracting COVID-19 before the vaccination, after the primary COVID-19 vaccine series, and after the booster dose resulted with a mean value of 7.7, 4.6, and 4.2, respectively. Females, those who had a lower mean self-perceived health status, who perceived COVID-19 as a very serious health problem, who decided to receive the booster dose because they perceived to be at risk of getting COVID-19, and who expressed interest in acquiring more information regarding the COVID-19 vaccine booster dose were more likely to report a higher concern of contracting COVID-19 after the booster dose. Over two-thirds were willing to respect the three main COVID-19 preventive measures following the booster dose. The multinomial logistic regression analysis showed that those who had used all three main preventive measures before and after the second or single dose were less likely to perceive COVID-19 as a serious illness. Those who had used them irregularly were less likely to have used official government organizations and scientific journals as sources of information. This survey provided an understanding regarding the adherence to COVID-19 preventive measures that may help to target policy interventions needed to reduce the spread of SARS-CoV-2.

## 1. Introduction

The novel coronavirus disease 2019 (COVID-19) pandemic has spread rapidly throughout the world and as 17 February 2022, over 414 million cases and 5 million deaths have been reported worldwide [1]. The Government of Italy had introduced numerous restrictive policies in an effort to control the spread of the infection, since it announced a national lockdown with the temporary suspension of several non-essential activities, indicated the mandatory adoption of several public health measures like keeping social distancing, washing hands frequently and thoroughly, wearing face-masks, and also recommended behaviors like avoiding crowded places, using public transport, and following quarantine rules [2].

In Italy, as in many other countries, the COVID-19 vaccination program nationwide started on 27 December 2020, and on 20 February 2022 high global vaccination rates have been achieved since 88.8% of population aged 12 years and over had received both doses and 91.1% had received at least one dose [3,4]. Moreover, the national protocol recommended, a booster dose of an mRNA vaccine to people of 18 years or older after completion of a primary COVID-19 vaccine series as of 1 December 2021, and as of 20 February 2022, 36 million doses have been administered to people of the same age group [4,5].

Vaccinated people and those who had received the COVID-19 vaccine booster dose, should continue to take all appropriate recommended precautions to protect themselves and others from the disease. To the best of our knowledge, the literature has neglected to investigate the change in plans to use preventive measures among those who had received a COVID-19 booster dose, and only a few studies have analyzed the compliance with the recommended prevention behaviors among vaccinated individuals [6,7,8,9]. Therefore, in this current epidemiological scenario, the present research offers an important contribution to the existing literature. Thus, the objectives of the present cross-sectional survey were to explore any changes over time in the adherence to COVID-19 preventive measures of vaccinated individuals in Italy. An additional objective was to identify the predictors associated with such changes.

## 2. Materials and Methods

### 2.1. Setting and Study Population

This survey, conducted between 16 and 26 November 2021, is part of a larger study conducted to explore the perceptions and practices toward COVID-19 vaccination and associated factors among different populations [10,11,12,13]. The sample consisted of all individuals aged 18 years and above who attended the immunization center of a teaching hospital located in Naples, in the South of Italy, to receive a COVID-19 vaccine booster dose. The minimum required sample size was estimated to be 481, with an assumption of a prevalence of 50% of subjects in the population having used all the three main COVID-19 public health measures (wearing a mask, hand washing, physical distancing) before receiving the first dose, following the second dose, and also who were willing to use them following the booster dose, a margin of error of 5%, a confidence interval of 95%, and an 80% response rate.

### 2.2. Procedures

The survey instrument and study protocol were approved by the Ethics Committee of the Teaching Hospital of the University of Campania “Luigi Vanvitelli”. All individuals were personally approached once they presented to the immunization center, by trained postgraduate students, and were offered the opportunity to voluntarily participate in the study. Potential participants were provided with detailed information about the nature and scope of the research, the methodology and the investigators, and told that their participation was on a voluntary basis and that anonymity, confidentiality, and the privacy of the data were guaranteed and protected, and that they had the right to withdraw from the research at any time for any reason. The postgraduate students handed over to the participants a printed copy of the survey and the completion, and return of the questionnaire constituted the informed consent to act as a participant in this research. Respondents did not receive any gift or monetary compensation for their participation.

### 2.3. Data Collection

The survey instrument, based upon previously used questionnaires in studies conducted by some of us [10,12,13], was pilot tested with 20 individuals who were not part of the study sample, for clarity of the items and also suggestions for improvement.

The data were collected by self-administration of a questionnaire that included into four parts. The first part was designed to collect participants’ socio-demographic background and general characteristics, including gender, age, marital status, level of education, number of children in home, number of persons in the household, occupation, underlying chronic medical conditions, having been infected with SARS-CoV-2, have had someone in the family or a friend test positive for COVID-19, self-perceived health status currently, and whether they had received the first and the second doses of the COVID-19 vaccine. The second part assessed participants’ attitude regarding COVID-19 infection and the vaccine booster dose with seven related statements, presented with a ten-point Likert scale for response, ranging from 1 = not at all to 10 = at all. The statements included the perceived personal risk of contracting COVID-19 before and after completion of the primary COVID-19 vaccine series and after the booster dose, concern about the efficacy and safety of the booster dose and about the severity of COVID-19, and trust in the information acquired about the booster dose. One item was used to assess specific motives for vaccination. In the third part participants were asked if they had done any of following ten activities before receiving the first dose and following the second dose (or single dose) of the COVID-19 vaccination and their willingness toward such activities following the booster dose: hand washing, maintaining physical distancing, wearing a mask, doing physical activity outdoors, eating at a restaurant, using public transport, attending indoor public places, greeting with a handshake and attending indoor and outdoor crowded places. These questions were answered as ‘yes’ or ‘no’ for the behavior and ‘yes’ or ‘no’ or ‘don’t know’ for the willingness. The fourth part queried participants’ sources of information related to vaccine booster doses against COVID-19 and whether they would like to receive additional information.

### 2.4. Statistical Analysis

First, descriptive statistics were used and data are expressed as relative frequency for categorical variables and mean and standard deviation for continuous variables. Second, a chi-square test and one-way analysis of variance (ANOVA) were used where appropriate to test for associations between the different outcomes of interest and each of the explanatory continuous and categorical variables, respectively. Third, variables with a *p*-value ≤ 0.25 in the bivariate analysis were adjusted in three multivariate linear and multinomial logistic regression models conducted to identify the factors associated with the predictive variables. The outcomes of the three multivariate models were the following: concern of contracting COVID-19 after the booster dose (Model 1), perception that COVID-19 is a very serious health problem (Model 2), and use of the three main COVID-19 public health measures (wearing a mask, hand washing, physical distancing) (Model 3). In Model 3, the outcome variable was coded such that those who use all the three main COVID-19 public health measures (wearing a mask, hand washing, physical distancing) before receiving the first dose, following the second dose (or single dose), and were willing to use following the booster dose, as reference category, were compared to (i) use before receiving the first dose and following the second dose, and (ii) irregular use. The following independent variables have been selected because they are potentially related to all outcomes: gender (male = 0; female = 1), age, in years (continuous), marital status (unmarried/separated/divorced/widowed = 0; married/cohabited with a partner = 1), baccalaureate/graduate degree (no = 0; yes = 1), healthcare worker (no = 0; yes = 1), having cohabitants (no = 0; yes = 1), having at least a chronic medical condition (no = 0; yes = 1), having been infected with SARS-CoV-2 (no = 0; yes = 1), have had friends or family members who were diagnosed with COVID-19 (no = 0; yes = 1), current self-perceived health status (continuous), having received information on the booster dose against COVID-19 from official government organizations and scientific journals (no = 0; yes = 1), and need of additional information on booster dose against COVID-19 (no = 0; yes = 1). Moreover, the variables regarding the number of children in home (0 = none; 1 = at least one) and perception that COVID-19 is a very serious health problem (continuous) were included in Models 1 and 3. Furthermore, the variable concerning having received the booster dose because they were perceived to be at risk of getting COVID-19 (0 = no; 1 = yes) was added in Model 1. A *p* = 0.2 and *p* = 0.4 were used to select candidate variables for retention and exclusion in the final multivariate linear regression models. A relative risk ratio (RRR) with a 95% confidence interval (CI) was calculated to estimate the strength of association in the multinomial logistic regression model, whereas standardized regression coefficients (ß) in the linear regression models were calculated. For all analyses, a two-tailed *p*-value equal to or less than 0.05 indicated statistical significance. All analyses were conducted in STATA version 15.1 [14].

## 3. Results

### 3.1. Characteristics of the Respondents

The entire cohort of individuals attending the immunization center during the study period included 824 subjects and a total of 795 participated in the study for a response rate of 96.5%.

The main characteristics of the survey participants are detailed in Table 1. The distribution of the sample revealed the predominance of participants female (60.4%), unmarried (76.2%), students (58.9%), the mean age was 32.9 years, the mean self-perceived health status was 8.6, only 10.8% had at least one chronic medical condition, only 4.9% reported having tested COVID-19 positive, and the vast majority reported that someone in the family or a friend had tested positive for COVID-19 (86.8%).

### 3.2. Attitude towards COVID-19

Regarding the participants’ attitudes, all measured on a 10-point Likert scale, the concern of contracting COVID-19 before the vaccination, after completion of the primary COVID-19 vaccine series, and after the booster dose, resulted with a mean value of 7.7, 4.6, and 4.2, respectively, with 29%, 1.7%, and 1.9% believing that they are at higher risk of acquiring the infection. The results of the multivariate linear regression analysis to identify the independent factors associated with two outcomes of interest are presented in Table 2. Five variables were identified to be significantly associated with the concern of contracting COVID-19 after the booster dose based on the multivariate linear regression analysis. Females, those who had a lower mean self-perceived health status, those who perceived COVID-19 as a very serious health problem, those who decided to receive the booster dose because they perceived to be at risk of getting COVID-19, and those who expressed their interest in acquiring more information regarding the COVID-19 vaccine booster dose were more likely to report a higher concern of contracting COVID-19 after the booster dose (Model 1). The mean values of the perceived concern over the efficacy of the booster dose and over its safety were 3.1 and 4.2, respectively. Individuals showed a high perception that COVID-19 was a very serious health problem with a mean value of 8.4 with more than one-third (34.6%) that was very concerned. The mean value of the trust in the information received about the booster dose was 7.7, with only 22.4% having this strongly positive attitude. The results of the multivariate linear regression analysis indicated that several independent variables significantly determined the higher overall mean score regarding the perception that COVID-19 was a very serious health problem. Female respondents, those married or cohabitating, who had a higher self-perceived health status, and those who relied on official government organizations and scientific journals as the main sources of information about the COVID-19 vaccine booster dose were more likely to perceive COVID-19 as a very serious health problem (Model 2).

The main motivating factor given by the respondents for having decided to receive the COVID-19 vaccine booster dose were as follows: self-protection (91.2%) and protection of their family members (88.7%) followed by being concerned about getting COVID-19 (50.7%), believing that COVID-19 is a severe disease (46.3%), and trusting in the efficacy of the booster dose (41.5%).

The results of comparing participants’ behaviors and attitude regarding different habits in all time points before receiving the first dose and following the second dose (or single dose) of the COVID-19 vaccination and willingness following the booster dose are presented in Table 3. From the period preceding the first dose to the period following the second dose there was an overall net increase in the proportion of individuals who participated in several activities that may increase the risk of catching and transmitting the virus. Indeed, the vaccination has changed the behavior and the largest increase in the risk post the second dose was for those who used public transport and ate at a restaurant with values moving from 34.2% to 70% and from 39.1% to 88.5%, respectively. There was also a decrease in the frequency of respondents who kept physical distancing to protect themselves before receiving the first dose (90.1%), compared to after the second dose (or single dose) (81.6%). Over two-thirds of the respondents (68.2%) were willing to respect the three main COVID-19 public health measures, such as wearing a mask, hand washing, and physical distancing, following the booster dose of the vaccination.

The results of the multinomial logistic regression analysis reported in Table 4 showed that respondents who had used all three main COVID-19 public health measures prior to receiving the first dose and following the second dose (or single dose) were less likely to report a higher perception that COVID-19 was a very serious health problem compared to those who have always used the three measures and were also willing to use them after the booster dose. Similarly, those who had used the three measures irregularly were less likely to have used official government organizations and scientific journals as main sources of information about the COVID-19 vaccine booster compared to respondents who have always used the three measures and were also willing to use them after the booster dose.

### 3.3. Sources of Information

Almost all interviewees declared actively searching for COVID-19 vaccine booster dose information (97.8%). Most respondents pointed to government health agencies (69.4%) as their principal source of knowledge, with multiple selections being allowed, followed by healthcare providers (56.9%), the Internet (36.9%), and mass-media (31.9%). Less than one third of the sample (28.3%) reported that they were interested in acquiring more information regarding the COVID-19 vaccine booster dose.

## 4. Discussion

To the best of our knowledge, this present survey is the first in Italy exploring the change over time in the adherence to COVID-19 preventive measures of vaccinated individuals and to consider the associated factors.

When participants were asked about their habits regarding the adherence to COVID-19 preventive measures, during a time period before receiving the first dose and following the second dose of the COVID-19 vaccination and their willingness following the booster dose, interestingly, a considerable decrease has been observed in the self-reported adherence with government recommendations when comparing the period of time preceding the first dose with the period following the second dose, whereas no particular differences have been observed between the second dose and the willingness after receiving the booster dose. More specifically, for a large number of participants, an increase of their activities in public places, such as eating at a restaurant, using transport, and attending indoor spaces, despite their role in the spread of the SARS-CoV-2, was observed. Compliance with two of the main COVID-19 public health measures, such as wearing a mask and hand washing, slightly increase after the second dose and decrease as a willingness after the booster dose, but what is more concerning is that maintaining physical distancing consistently decreased, from 90.1% to 74.7%, and hand hygiene was more used than physical distancing. In line with the present finding related to the decrease of the compliance, several previous studies using different samples of adults conducted within the past year found that the vaccination leads participants to reduce their compliance with public health measures [6,8,15]. Moreover, studies conducted in Italy showed a decrease over time in the behavior or in attitudes towards the adoption of the majority of the protective behaviors towards COVID-19 [13,16]. The finding of the present study should be interpreted with the fact that respondents may perceive a lower risk of contracting the virus when they are vaccinated. However, maintaining compliance with personal preventive measures still plays an important role in achieving the pandemic control among vaccinated individuals.

Respondents were asked about their preferred sources to keep themselves informed about the COVID-19 vaccine booster dose, and they indicated that they had used government health agencies and physicians to gather information. The influence of the different sources of information on the adherence to COVID-19 preventive measures and on the attitudes was investigated. It is important to highlight that a significant association has been observed, since respondents who used the three main COVID-19 public health measures (wearing a mask, hand washing, and physical distancing) irregularly did not rely on information on the COVID-19 vaccine booster dose from official government organizations and scientific journals, whereas those perceiving that COVID-19 was a severe disease were more likely to have received information from these sources. Availability and accessibility to information on COVID-19 vaccination are crucial for the general population to help the individuals to make informed decisions and to have safe preventive behaviors. The positive effect of the information received from institutional and scientific sources on the correct perception of the severity of COVID-19 aligns well with the results from previous research which found that the exposure to these sources have a positive impact on the level of knowledge, on the attitudes, and on the acceptability of the vaccination than those exposed to other sources [12,17,18,19,20,21]. Moreover, it was particularly notable that the internet has become the third major source for respondents seeking vaccine information. This is of partial concern since this source can vary in reliability and to assess the quality and relevance of online information about the vaccine’s general efficacy and safety is a difficult task for laypersons. Therefore, government health agencies and physicians should be conscious of their important role in the endeavor to better inform individuals and give them specific assistance with regard to which websites to use.

It is interesting to observe that the two most reported reasons why the current sample was receiving the COVID-19 vaccine booster dose related to their desire to protect themselves or their family members from being infected with COVID-19. Several of the most prevalent reasons for COVID-19 vaccination identified in this survey have been widely recognized in similar previous studies as strong drivers for vaccine uptake [22,23,24,25]. It is of concern to note that only half and less than half of the study participants indicated the perception of the risk of getting COVID-19 and that COVID-19 is a severe disease and that they trust in the efficacy of the booster dose, respectively. However, although the concept of risk perception is based on the understanding that an individual is more likely to take preventive measures when he or she believes themselves to be personally at risk of getting COVID-19, public health interventions are important, aiming at encouraging the adoption of healthier behavior. Additionally, the finding that COVID-19 severity and vaccine efficacy do not seem to motivate individuals to get vaccinated despite the extraordinary impact of the disease and the mass vaccination campaign at the time when this survey was conducted, clearly reveals a lack of information about the disease and the possible complications which make the vaccination necessary. Therefore, public health messaging and education programs could be more focused on the impact of diseases and are needed to make people aware of the fact that, although vaccinated, they are still at risk of getting the disease as well as reassuring them of the safety of the vaccination and also helping them understand the impact of the vaccination on SARS-CoV-2 spread, which may increase the likelihood of compliance.

Multivariable linear and multinomial logistic regression analyses were conducted to examine predictors of the different outcomes of interest. Among the socio-demographic characteristics, gender was one of the strongest statistically independent influencers on attitudes in this study. Indeed, women were more likely to perceive COVID-19 as a very serious health problem and to report a higher concern of contracting COVID-19 after the booster dose than men. The higher concern is difficult to explain since in Italy, as in different parts of the world, the spread of the COVID-19 and the related mortality have hit males more than females. However, published literature together with ours showed gender differences, since females were more concerned about COVID-19, experienced higher levels of fear of COVID-19, and were more likely to accept the vaccine against SARS-CoV-2 [24,26,27,28,29,30,31]. Moreover, the perceived severity of COVID-19 was a key factor in using all three main COVID-19 public health measures, since respondents who received the first dose and (or single dose) were less likely to report concern following the second dose compared to those who have always used the three measures and were also willing to use them after the booster dose. The finding regarding the perceived severity of COVID-19 infection is consistent with results from previous studies showing that this belief was associated with the adherence to COVID-19 preventive measures or with the willingness to receive this vaccination [10,12,32,33,34]. This is of great concern given the well-known epidemiological impact of the disease with the related serious complications and death and the crucial role of these preventive measures at the individual level for the reduction of the risk of infection.

Of course, it is important to note that in drawing the conclusions of this survey, some potential methodological limitations should be considered. First, this survey adopted a cross-sectional research design, which makes it challenging to establish the causality inference between the different outcomes of interest and the factors. Second, data collection was at a single immunization center and any generalization to populations in other regions of Italy from the results of this study should be made with caution. Third, due to the role that the self-reported nature of the collected information potential recall bias and social desirability bias may have played in some data, the responses may partly depend on the respondents’ sincerity, which could result in either underreporting or overreporting of behavior/attitudes in order to appear a certain way and to avoid criticism. All responses were confidential and anonymous in order to respondents, and this may have positively affected the honesty of the responses. Despite the above limitations, this survey offers a snapshot depiction of the vaccinated individuals changes over time regarding the adherence to COVID-19 preventive measures and the associated factors.

In conclusion, this survey of a large sample of vaccinated individuals in Italy provided a foundational understanding regarding the adherence to COVID-19 preventive measures in the context of a large infectious disease pandemic such that it may help to target policy interventions that are still needed to reduce the spread of SARS-CoV-2.

## Figures and Tables

**Table 1:** Socio-demographic and key characteristics of the study population.

Characteristics	N	%
Age, years	32.9 ± 12.5 (20–88) *	
Gender		
Female	476	60.4
Male	312	39.6
Marital status		
Unmarried/separated/divorced/widowed	606	76.2
Married/cohabited with a partner	189	23.8
Educational level		
High school degree or less	507	63.8
Baccalaureate/graduate degree	288	36.2
Number of children in home		
0	653	82.1
≥1	142	17.9
Number of cohabitants		
0	49	6.2
1–3	548	68.9
>3	198	24.9
Role		
Student	468	58.9
Healthcare worker	181	22.8
Other	145	18.3
Having at least a chronic medical condition		
No	709	89.2
Yes	86	10.8
Having been infected by SARS-CoV-2		
No	756	95.1
Yes	39	4.9
Having friends or family members who were diagnosed with COVID-19		
No	105	13.2
Yes	690	86.8
Self-rated global health status	8.6 ± 1.2 (2–10) *	
Self-rated global health status after the first dose of the COVID-19 vaccination	8.2 ± 1.5 (2–10) *	
Self-rated global health status after the second dose of the COVID-19 vaccination	7.8 ± 1.8 (1–10) *	

* Mean ± Standard deviation (range).

**Table 2:** Predictors of the first two outcomes of interest using multivariate linear regression analysis.

Variable	Coeff.	t	*p*
**Model 1. Concern of contracting COVID-19 after the booster dose** **F (12,769) = 8.51, *p* < 0.0001, R^2^ = 11.7%, Adjusted R^2^ = 10.3%**			
Perception of COVID-19 as a very serious health problem	0.26	5.05	<0.001
Females	0.48	2.89	0.004
Interest in acquiring more information regarding the booster dose Having decided to receive the booster dose because they perceived to be at risk of getting COVID-19	0.44	2.50	0.013
	0.50	2.31	0.021
Lower mean self-perceived health status	−0.13	−1.96	0.05
Having been infected with SARS-CoV-2	0.60	1.67	0.095
Married/Cohabitating	0.38	1.55	0.12
Healthcare workers	0.30	1.54	0.13
Not having had friends or family members who were diagnosed with COVID-19	−0.35	−1.49	0.14
Not having received information on booster dose against COVID-19 from official government organizations and scientific journals	−0.22	−1.19	0.23
Older	0.01	1.09	0.28
Having at least a chronic medical condition	0.26	1.02	0.31
**Model 2. Perception that COVID-19 is a very serious health problem** **F (7,777) = 11.14, *p* < 0.0001, R^2^ = 9.1%, adjusted R^2^ = 8.3%**			
Females	0.74	6.55	<0.001
Married/Cohabitating	0.59	4.36	<0.001
Higher mean self-perceived health status	0.13	2.83	0.005
Having received information on booster dose against COVID-19 from official government organizations and scientific journals	0.27	2.12	0.034
Having had friends or family members who were diagnosed with COVID-19	0.32	1.92	0.055
Healthcare workers	0.24	1.82	0.07
Interest in acquiring more information regarding the booster dose against COVID-19	0.18	1.47	0.14

**Table 3:** Respondents’ behaviors regarding different habits before the vaccination, after completing the vaccination course, and willingness after receiving the booster dose of the vaccination.

Habits	Behavior before the Vaccination		Behavior after Completing the Vaccination Course		Willingness after Receiving the Booster Dose	
	*n*	%	*n*	%	*n*	%
Hand washing	743	93.5	757	95.3	730	92.3
Maintain physical distancing	716	90.1	649	81.6	590	74.7
Wearing a mask	709	89.2	777	97.7	748	94.3
Doing physical activity outdoors	516	65.8	593	75.7	567	72.2
Eating at a restaurant	309	39.1	697	88.5	697	88.2
Using public transport	270	34.2	552	70	563	71.1
Attending indoor public places	214	27.2	521	66.4	499	63.4
Greeting with a handshake	159	20.1	292	37	298	37.6
Attending indoor and outdoor crowded places	140	17.6	315	39.8	310	39.3

**Table 4:** Predictors of the third outcome of interest using multinomial logistic regression analysis.

Model 3. Adherence to the Three Main COVID-19 Public Health Measures (Wearing a Mask, Hand Washing, Physical Distancing)						
Variable	Use before Receiving the First Dose and Following the Second Dose			Use Irregularly		
	RRR	95% CI	*p*	RRR	95% CI	*p*
Perception of COVID-19 as a very serious health problem	0.86	0.74–0.99	0.044	0.91	0.82–1.01	0.07
Having received information on booster dose against COVID-19 from official government organizations and scientific journals	0.59	0.34–1.03	0.07	0.55	0.39–0.78	0.001
Healthcare workers	1.71	0.83–3.52	0.14	1.25	0.78–2.01	0.35
Baccalaureate/graduate degree	0.91	0.47–1.77	0.78	0.70	0.46–1.05	0.09
Interest in acquiring more information regarding the booster dose	1.08	0.61–1.92	0.79	1.38	0.99–1.93	0.06

## Data Availability

The data presented in this study are available on request from the corresponding author.
